# Microsurgical Transplantation of Pedicled Muscles in an Isolation Chamber—A Novel Approach to Engineering Muscle Constructs via Perfusion-Decellularization

**DOI:** 10.3390/jpm12030442

**Published:** 2022-03-11

**Authors:** Aijia Cai, Zengming Zheng, Wibke Müller-Seubert, Jonas Biggemann, Tobias Fey, Justus P. Beier, Raymund E. Horch, Benjamin Frieß, Andreas Arkudas

**Affiliations:** 1 Department of Plastic and Hand Surgery and Laboratory for Tissue Engineering and Regenerative Medicine, University Hospital of Erlangen, Friedrich-Alexander University of Erlangen-Nürnberg (FAU), Krankenhausstraße 12, 91054 Erlangen, Germany; zhengzengming@163.com (Z.Z.); wibke.mueller-seubert@uk-erlangen.de (W.M.-S.); raymund.horch@uk-erlangen.de (R.E.H.); bennifriess@gmx.de (B.F.); andreas.arkudas@uk-erlangen.de (A.A.); 2 Department of Materials Science and Engineering, Institute of Glass and Ceramics, Friedrich-Alexander-University Erlangen-Nürnberg (FAU), Martensstr. 5, 91058 Erlangen, Germany; jonas.biggemann@fau.de (J.B.); tobias.fey@fau.de (T.F.); 3 Frontier Research Institute for Materials Science, Nagoya Institute of Technology, Gokiso-cho, Showa-ku, Nagoya 466-8555, Japan; 4 Department of Plastic Surgery, Hand Surgery, Burn Center, University Hospital RWTH Aachen, Pauwelsstr. 30, 52074 Aachen, Germany; jbeier@ukaachen.de

**Keywords:** muscle transplantation, rat gastrocnemius, in situ stimulation, muscle contraction, perfusion-decellularization

## Abstract

Decellularized whole muscle constructs represent an ideal scaffold for muscle tissue engineering means as they retain the network and proteins of the extracellular matrix of skeletal muscle tissue. The presence of a vascular pedicle enables a more efficient perfusion-based decellularization protocol and allows for subsequent recellularization and transplantation of the muscle construct in vivo. The goal of this study was to create a baseline for transplantation of decellularized whole muscle constructs by establishing an animal model for investigating a complete native muscle isolated on its pedicle in terms of vascularization and functionality. The left medial gastrocnemius muscles of 5 male Lewis rats were prepared and raised from their beds for in situ muscle stimulation. The stimulation protocol included twitches, tetanic stimulation, fatigue testing, and stretching of the muscles. Peak force, maximum rate of contraction and relaxation, time to maximum contraction and relaxation, and maximum contraction and relaxation rate were determined. Afterwards, muscles were explanted and transplanted heterotopically in syngeneic rats in an isolation chamber by microvascular anastomosis. After 2 weeks, transplanted gastrocnemius muscles were exposed and stimulated again followed by intravascular perfusion with a contrast agent for µCT analysis. Muscle constructs were then paraffin embedded for immunohistological staining. Peak twitch and tetanic force values all decreased significantly after muscle transplantation while fatigue index and passive stretch properties did not differ between the two groups. Vascular analysis revealed retained perfused vessels most of which were in a smaller radius range of up to 20 µm and 45 µm. In this study, a novel rat model of heterotopic microvascular muscle transplantation in an isolation chamber was established. With the assessment of in situ muscle contraction properties as well as vessel distribution after 2 weeks of transplantation, this model serves as a base for future studies including the transplantation of perfusion-decellularized muscle constructs.

## 1. Introduction

Tissue engineering of skeletal muscle holds great promise for the treatment of volumetric muscle loss. It can help to circumvent substantial donor site morbidity, resulting from donor tissue transfer, including free autologous muscle flaps [1,2,3]. Common tissue engineering approaches have attempted to create three-dimensional (3D) constructs by combining scaffolds with stem cells and growth factors [3,4]. However, the production of biocompatible and stable scaffolds can be time consuming and expensive and cell adherence, invasion, and differentiation can be demanding in this synthetic 3D environment [5,6].

Decellularized extracellular matrix scaffolds have been a popular platform for regenerating skeletal muscle, as they contain structural proteins and molecules of skeletal muscle tissue, which is difficult to mimic by artificial means [6,7]. Established protocols have mostly applied diffusion techniques on skeletal muscle so far, although perfusion-decellularization through a vascular pedicle seems more beneficial by retaining the complex 3D architecture of the native tissue while effectively removing all cells [8]. The presence of a vascular pedicle enables subsequent transplantation of the decellularized and possibly recellularized construct in vivo.

Critical size defects necessitate volumetric tissue constructs for reconstruction, which on the other hand depend on adequate blood supply for survival [9]. Thus, a functional blood vessel network is a prerequisite for the growth of tissues and organs in vivo. The arteriovenous (AV) loop model contains a vein graft anastomosed to an artery and a vein, creating a loop that is transferred to an enclosed implantation chamber [10]. This creates an isolated microenvironment, which is connected to the living organism only by means of the vascular axis. Any disturbing influences from the surroundings are shielded off and the tissue with its vascularization can be analyzed in the controlled environment. 

Perfusion-decellularization of a whole muscle via its main vascular pedicle, subsequent recellularization of the skeletal muscle matrix, and transplantation of the whole construct in vivo have the potential to generate new functional muscle tissue. An isolation chamber allows for analysis of the newly generated and vascularized tissue independent of external factors similar to the AV-loop model. Prior to establishing such a model, baseline values concerning vascularization and functionality of such transplanted muscle constructs are needed.

The principal contribution of this work is a novel in vivo animal model for investigating a transplanted complete native muscle isolated on its pedicle in terms of vascularization and functionality.

## 2. Methods

### 2.1. Experimental Animals

Animal experiments were carried out following the German regulations for the care of laboratory animals at all times. Experiments were approved by the local Animal Care Committee (approval number RUF 55.2.2-2532.2-658-48). 

Gastrocnemius muscles were taken from the left hindlimb of 5 male Lewis donor rats (Charles River Laboratories, Sulzfeld, Germany) and transplanted into the left hindlimb of 5 syngenic recipient rats. 

All animals were anesthetized during the surgical procedures through the administration of gaseous isoflurane (op-pharma, Burgdorf, Germany) at concentrations between 1.5–5% under spontaneous breathing. For analgesia, animals received intravenous meloxicam (2 mg/kg) and butorphanol (1.5 mg/kg), whereas butorphanol was substituted every 2 h. 

### 2.2. Muscle Preparation

**Figure 1:**
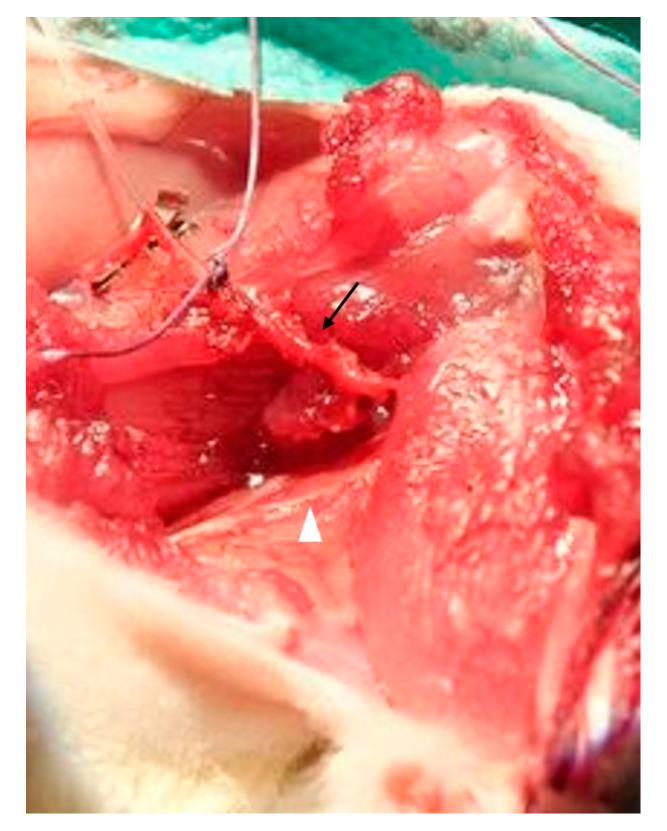
After surgical preparation, the gastrocnemius muscle was attached in situ via its vascular pedicle (arrow) and the tibial nerve (white arrowhead).

**Figure 2:**
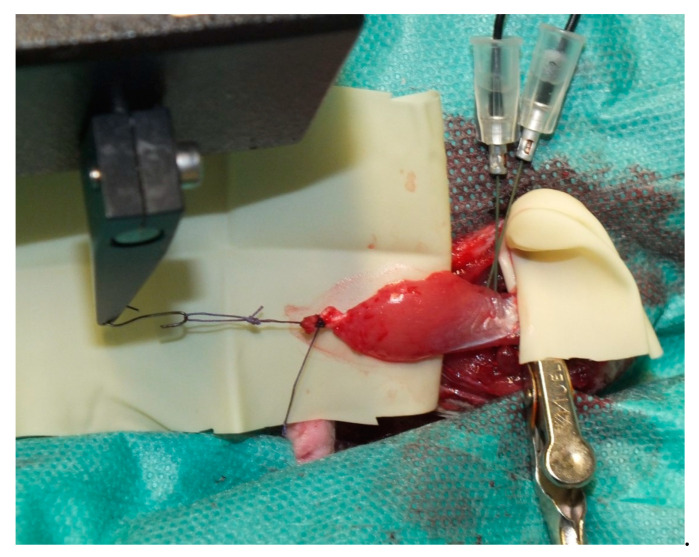
For electrical stimulation, the muscle was connected to a servomotor lever arm distally and fixed with a clamp proximally. Electrodes ware placed near the entry of the tibial nerve.

Donor animals were placed in the supine position on a heating plate. Dissection through the adductor muscles of the left hind limb was carried out under aseptic conditions until the medial gastrocnemius muscle was reached and freed from all muscular and tendon attachments. All vessels branching off the muscle except for the popliteal artery and vein, representing the main pedicle, were ligated. Popliteal vessels were tracked proximally up to their origin from the femoral vessels until the gastrocnemius muscle was solely attached in situ via its vascular pedicle and the tibial nerve (Figure 1). The distal tendon was then tied with a 4-0 Vicryl suture (Ethicon, Somerville, NJ, USA) and connected to a servomotor lever arm (model 305C Dual-Mode Lever Arm System; Aurora Scientific Inc, Aurora, Ontario, Canada), while the proximal tendon was fixed in a clamp on a self-constructed metal frame, placed under the heating plate (Figure 2). After stimulation of the gastrocnemius, the rat receiving the gastrocnemius muscle as a transplant was surgically prepared (see Section 2.4 for details). 

### 2.3. Muscle Stimulation

Prior to transplantation and perfusion, the gastrocnemius muscle was stimulated via electrodes placed into the muscle close to where the tibial nerve enters the muscle. A stimulation and recording system (150 A and 615A Dynamic Muscle Control and High-Throughput Analysis software suite; Aurora Scientific Inc., Aurora, ON, Canada), was used to stimulate the muscle after optimal length was determined as described by Tamayo et al. [11]. For twitches, the muscle was stimulated every 3 s with 1 ms pulses for 10 repetitions. The maximum force (i.e., peak force), maximum rate of contraction and relaxation, time to maximum contraction and relaxation, and maximum contraction and relaxation rate were measured. This was followed by 80 Hz, 100 Hz, and 120 Hz tetanic stimulation. Peak force, maximum contraction rate, and time to maximum contraction were assessed. For fatigue testing, a 150 Hz burst of stimulation was applied to the muscle every 3 s for 7.5 min. The fatigue index was determined as the ratio between the maximum and minimum force difference and the maximum force. A break of at least 1 min was taken between each measurement. For passive tension properties, the muscle was stretched to 110% of its original length in 1% steps. During each stretch, contractile forces to gain the length of the muscle were measured. 

### 2.4. Muscle Transplantation

After stimulation of the donor rat muscle, a second rat was prepared for surgery in the supine position. The left hind limb was incised and the saphenous artery and superficial inferior epigastric vein were dissected and prepared for vascular anastomosis. 

**Figure 3:**
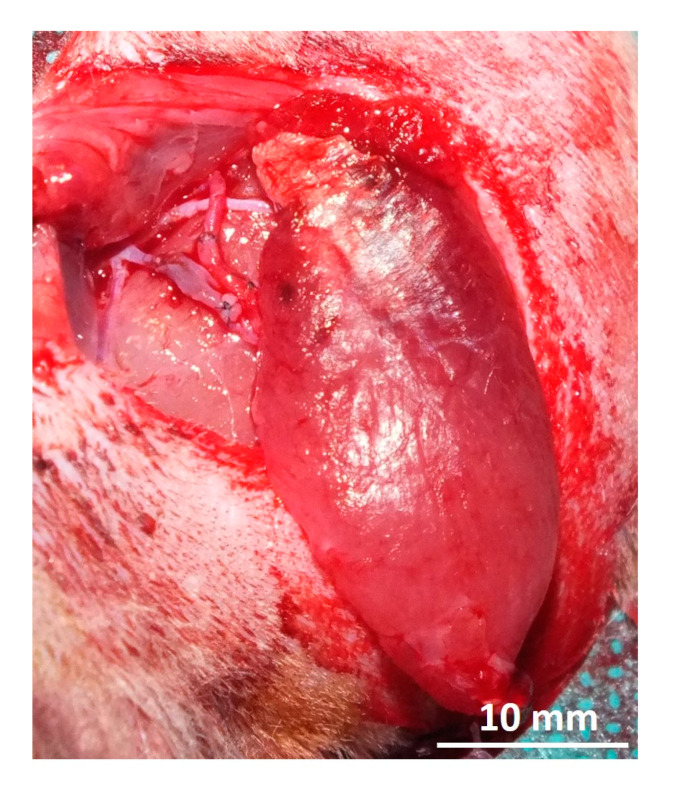
Gastrocnemius muscle was transplanted via anastomosis of the femoral artery to the saphenous artery and femoral vein to the superficial inferior epigastric vein.

**Figure 4:**
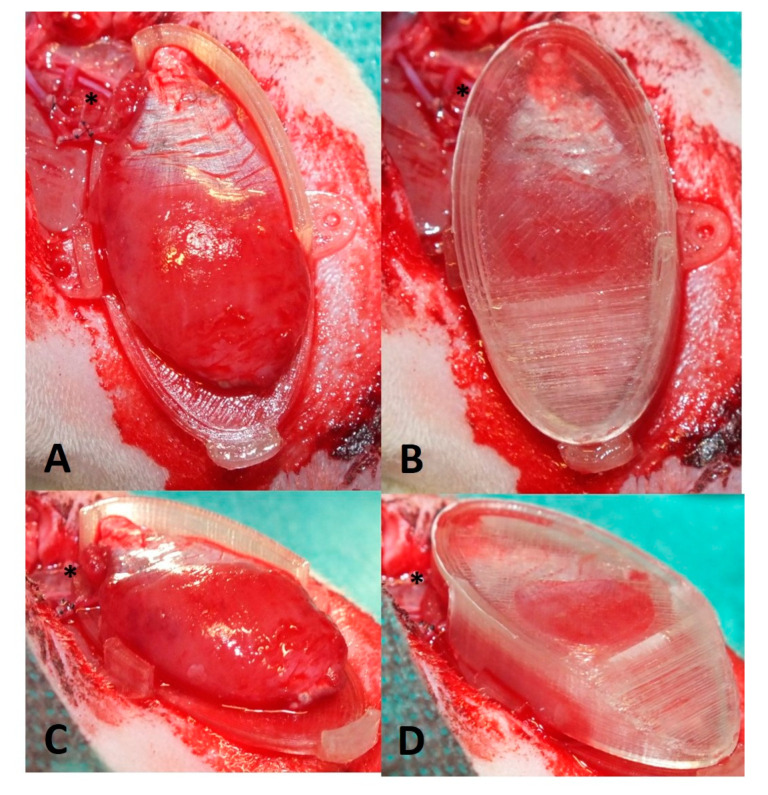
Different perspectives showing transplanted gastrocnemius muscle placed onto bottom part of isolation chamber (**A**,**C**) and into isolation chamber with lid on top (**B**,**D**). Note the opening of the chamber for the vascular pedicle (asterisk).

The dissected gastrocnemius muscle was harvested from the donor animal by cutting the femoral vessels. The muscle was flushed with heparine (50 I.U.) via the femoral artery until a clear heparine solution came out of the femoral vein. The wet weight of the muscle was determined. The donor rat was sacrificed and the gastrocnemius muscle was transplanted into the recipient rat by anastomosing the femoral artery to the saphenous artery and femoral vein to the superficial inferior epigastric vein (SIEV) with 11-0 interrupted sutures (Figure 3). The patency of anastomoses was confirmed via the milking patency test [12]. The revascularized muscle was placed into an isolation chamber made out of polylactide via 3D printing by fused deposition modeling (FDM) using an Ultimater 2+ (Ultimaker B.V., Utrecht, The Netherlands). The isolation chamber consisted of two parts: one bottom part, which was sutured to the anterior muscle of the hind leg (Figure 4A,C), and a lid that enclosed the transplanted muscle while avoiding squeezing of the muscle (Figure 4B,D). Anastomoses were fixed with fibrin glue to avoid kinking of the vessels. The skin was closed in 2 layers. Antibiotics (7.5 mg/kg enrofloxacin) were administered perioperatively intravenously and continued subcutaneously for 5 days. Animals received 10 mg/kg low molecular weight heparin and meloxicam (2 mg/kg) subcutaneously for 5 days.

### 2.5. Perfusion and Explantation of Constructs

**Figure 5:**
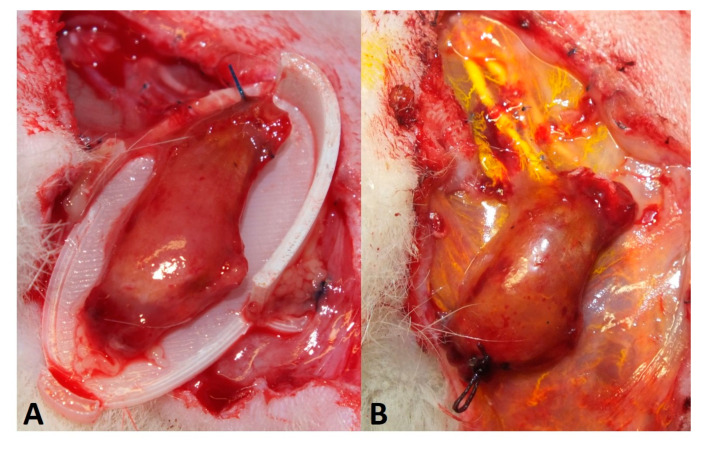
After 2 weeks of implantation, the gastrocnemius muscle has shrunken to approximately half its original size (**A**). After electrical stimulation, the muscle was perfused with a contrast agent (Microfil^®^ = yellow) (**B**).

After two weeks of implantation, rats were again anesthetized as described above and placed in the supine position. The left hind limb was dissected and the isolation chamber was opened to expose the transplanted gastrocnemius muscle (Figure 5A). The muscles were taken out of the chamber and stimulation was carried out as described above. After that, muscles were perfused with the contrast agent Microfil^®^ as described previously [13]. Briefly, laparotomy incision was performed and animals were perfused by flushing the aorta with 150 mL of heparine solution and afterward with 20 mL yellow Microfil^®^ (MV-122) containing 2.5% of MV Curing Agent (both Flowtech, Carver, MA, USA) (Figure 5B). Finally, the aorta and inferior vena cava were ligated, and the rats were placed at 7 °C for 24 h. Constructs were explanted in toto and fixed in 3.5% formalin solution overnight and placed in PBS solution for microtomography (µCT) analysis. 

### 2.6. Micro-Computed Tomography

High-resolution µCT scans were performed using Skyscan 1172 with an 11-MP detector and a tungsten tube at a voltage of 80 kV and a current of 100 mA (Skyscan B.V., Leuven, Belgium) as described previously [14]. Briefly, after scanning at 180° with a rotation step of 0.25° and a resolution of 4.47 µm/voxel, the data were reconstructed with Radeon back Transformation using the tomographic reconstruction software (NRecon Client and Server 1.7.42 with GPU support; Skyscan Leuven, Belgium) while adjusting the X/Y shift and alignment during measurement. The visualization and vessel radius evaluation was carried out by imaging software (Amira 2021.1; Thermo Fisher Scientific, Berlin, Germany). 

### 2.7. Immunohistochemical Analysis

After µCT analysis, constructs were processed for paraffin embedding. The feasibility of histologic analysis after Microfil^®^ perfusion has been demonstrated before [14,15,16]. Three-micrometer cross-sections were obtained from the middle of the gastrocnemius muscles perpendicular to the main vascular axis with a microtome (Leica Microsystems, Wetzlar, Germany). Hematoxylin and eosin (HE) were performed according to standard protocols. For myosin heavy chain (MHC) staining, sections were blocked with 10% goat serum (Vector Laboratories, Burlingame, CA, USA) and incubated with anti-fast myosin skeletal heavy chain (MHC) antibody (ab91506, Abcam, Cambridge, UK) at a concentration of 5 µg/mL overnight at 4 °C. After washing with TBS-T (1 mL Tween20 per 1 L of 100 mM Tris and NaCl in H_2_O, pH 7.4), Alexa Fluor 647 goat anti-rabbit IgG (H + L) cross-adsorbed secondary antibody (A-21244) was used at a concentration of 4 µg/mL for 30 min at room temperature. Probes were counterstained with DAPI 1 µg/mL (diamidine-phenylindole-dihydrochloride, Thermo Fisher Scientific Inc.) for 5 min. For visualizing neuromuscular junctions, deparaffinized sections were stained with recombinant anti-Synaptophysin antibody [YE269] (ab32127, Abcam) at a concentration of 0.5 µg/mL and incubated overnight. Afterwards, sections were incubated with Alexa Fluor 488 anti-rabbit (A11008, Thermofisher Scientific Inc.) at a concentration of 4 µg/mL for 30 min followed by α-Bungarotoxin, Alexa Fluor 647 conjugated (B35450, Thermo Fisher Scientific Inc., Waltham, MA, USA) for 1 h and counterstaining with DAPI as described above. For macrophage detection, CD68 staining was performed with anti-CD68 antibody (BIO-RAD, Hercules, CA, USA) in a dilution of 1:300 overnight on deparaffinized and blocked sections. For enzymatic detection, an alkaline phosphate-labeled anti-mouse-antibody and Fast Red TR/Naphthol AS (Sigma-Aldrich, Missouri, USA) were applied. Haemalaun was used for counterstaining. 

### 2.8. Vessel Quantification

Reconstructed two-dimensional cross-sections from µCT scans were exported as 256 grey value level images and used for segmentation of the vessels, as previously described [14]. Additional shrink and grow image calculation operations were carried out to ensure filled blood vessels for analysis to prevent misleading results from e.g., bubbles in the Microfil^®^. Accumulated vessel lengths were calculated for each vessel radius starting from 5 µm at histogram class widths of 10 µm. To exclude material that did not belong to the muscle, the cut-off was set at 335 µm. This agrees with the maximum diameter of 500–600 µm of the vascular pedicle of the gastrocnemius muscle. Values are summarized for every radius range of 40 µm, e.g., 5–45 µm. 

HE stained sections were used for vessel counting in ImageJ 1.53e (National Institutes of Health, Bethesda, MD, USA). After setting a scale, the lumen of all vessels with a clearly visible lumen or lumen filled with Microfil^®^ was outlined using the freehand tool in the ROI manager. The radius was calculated using the resulting area of the vessel lumen, and the number of radius ranges of 20 µm, e.g., 0.1–20 µm, 20.1–40 µm was depicted. 

### 2.9. Statistical Analysis

Data are expressed as the mean + standard deviation (SD). Data normality of force values was verified by the Shapiro–Wilk test. Pairwise comparisons between muscles before and after transplantation were carried out using paired *t*-test or Mann–Whitney test, as appropriate. Statistical analysis was performed using GraphPad Prism version 8.3, La Jolla, CA, USA. A *p*-value ≤ 0.05 was considered statistically significant.

## 3. Results

### 3.1. Surgery and Animals

All animals tolerated surgeries well. Weight properties of the involved rats and muscles with the corresponding ischemia time are listed in Table 1. 

Four out of 5 animals developed postoperative seroma, that were punctured once (2 animals) or twice (2 animals) under sterile conditions. At the time of explantation after 2 weeks, all muscles were vital but had shrunken to approximately half their original size (Figure 5A).

### 3.2. Muscle Function

**Figure 6:**
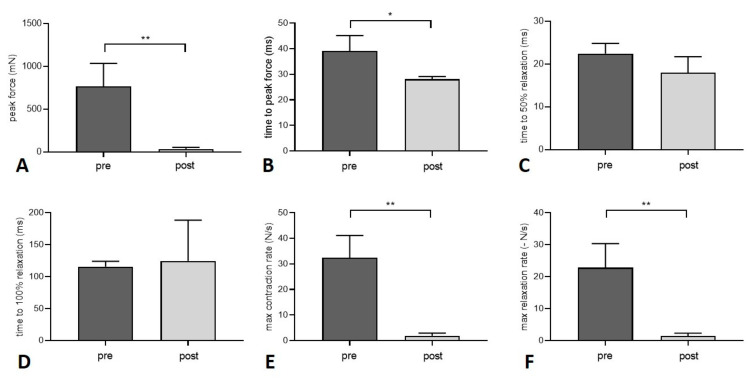
Force values before and 2 weeks after transplantation of the gastrocnemius muscle. Peak force (**A**), time to peak force (**B**), and maximum contraction and relaxation rate (**E**,**F**) decreased after transplantation while time to 50% and 100% relaxation (**C**,**D**) did not change (paired *t*-test). Level of significance was * *p* < 0.05, ** *p* < 0.01.

**Figure 7:**
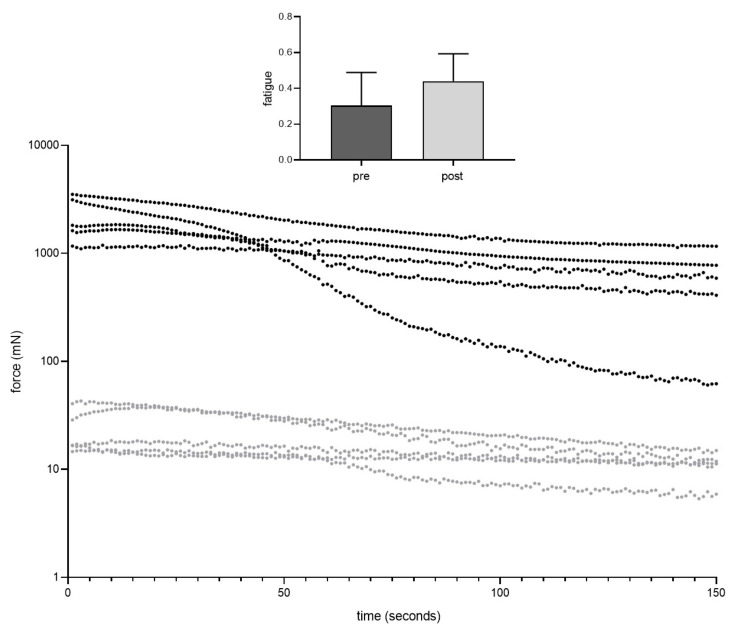
Fatigue index was similar before (dark grey) and 2 weeks after muscle transplantation (light grey) (paired *t*-test).

Peak force after twitch decreased significantly by 95.5% 2 weeks after the gastrocnemius muscle was transplanted within the isolation chamber (*p* = 0.0041) (Figure 6). Time to peak force, maximum contraction, and relaxation rate all decreased after transplantation (*p* = 0.011, *p* = 0.0018, and *p* = 0.0042, respectively). Time to 50% and 100% relaxation did not differ between the two groups (*p* = 0.0735 and *p* = 0.7621, respectively) (Figure 7). Peak force and contraction rate after tetanic stimulation decreased after transplantation for all frequencies (*p* = 0.0014 and *p* = 0.001, respectively, for 80 Hz, *p* = 0.0028 and *p* = 0.0003, respectively, for 100 Hz, *p* = 0.0058 and *p* = 0.0035, respectively, for 120 Hz). Time to peak force decreased at 80 Hz (*p* = 0.0115), 100 Hz (*p* = 0.0035), and 120 Hz (*p* < 0.0001). Detailed values for twitch and tetanus are listed in Table 2. 

**Figure 8:**
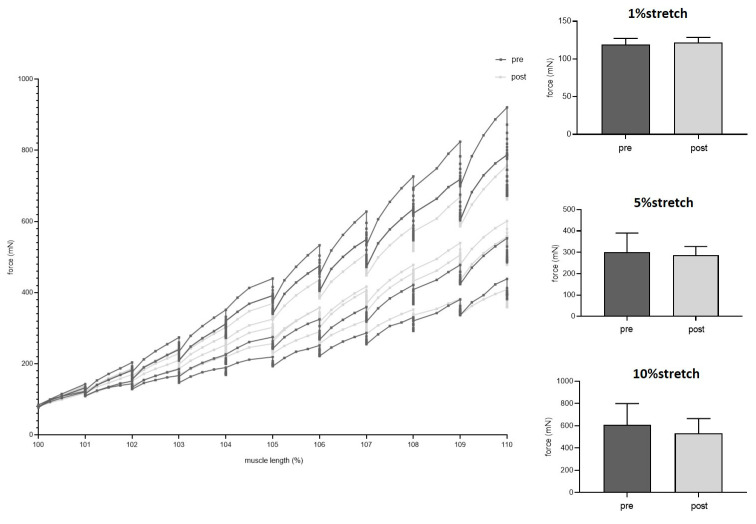
Passive stretch properties were similar before (dark grey) and 2 weeks after muscle transplantation (light grey). Exemplary data are shown for forces needed for 1%, 5%, and 10% length gain (paired *t*-test).

During fatigue testing, there was an obvious difference between pre and post transplantation groups (Figure 7). Nevertheless, fatigue index was similar between the two groups (*p* = 0.287). Passive stretch properties did not change after transplantation (Figure 8). For each % increase in length from 100–110%, contractile forces needed to maintain the respective length of the muscle did not differ between the two groups: 1% (*p* = 0.641), 2% (*p* = 0.707), 3% (*p* = 0.878), 4% (*p* = 0.832), 5% (*p* = 0.708), 6% (*p* = 0.631), 7% (*p* = 0.550), 8% (*p* = 0.437), 9% (*p* = 0.395), 10% (*p* = 0.323). 

### 3.3. Vessel Quantification

**Figure 9:**
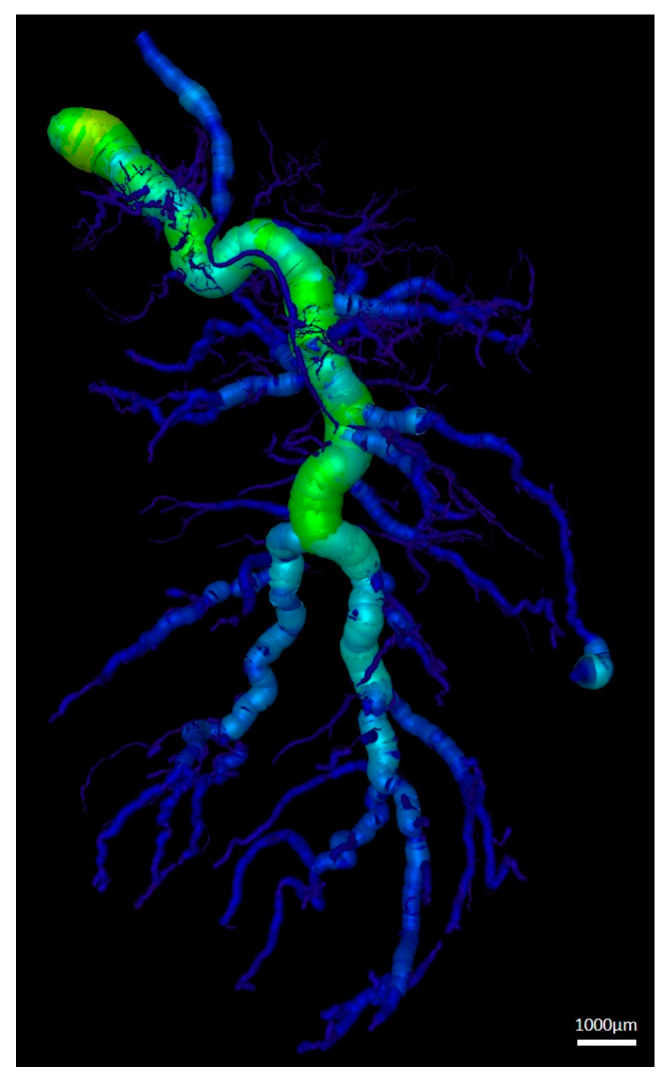
Exemplary 3D µCT scan of a gastrocnemius muscle perfused with contrast agent after 2 weeks of transplantation.

Three-dimensional (3D) µCT scans showed the cumulative lengths for different vessel radius ranges (Table 3 and Figure 9). Vessels with smaller radii (measuring 5–45 µm and 55–95 µm) were the highest in number and cumulative length. The larger the radius of the vessels the lower was the cumulative length.

Vessel analysis of immunohistochemical sections revealed a similar trend (Table 4): the smallest vessels, ranging from 0.1–20 µm, were the most prevalent while vessel radius ranges of 120.1–140 µm, 140.1–160 µm, and 240.1–260 µm were present in only 1 out of 5 muscles (rat 3 and rat 5, respectively).

### 3.4. Nerve-Muscle-Junction and Myogenic Marker Expression

**Figure 10:**
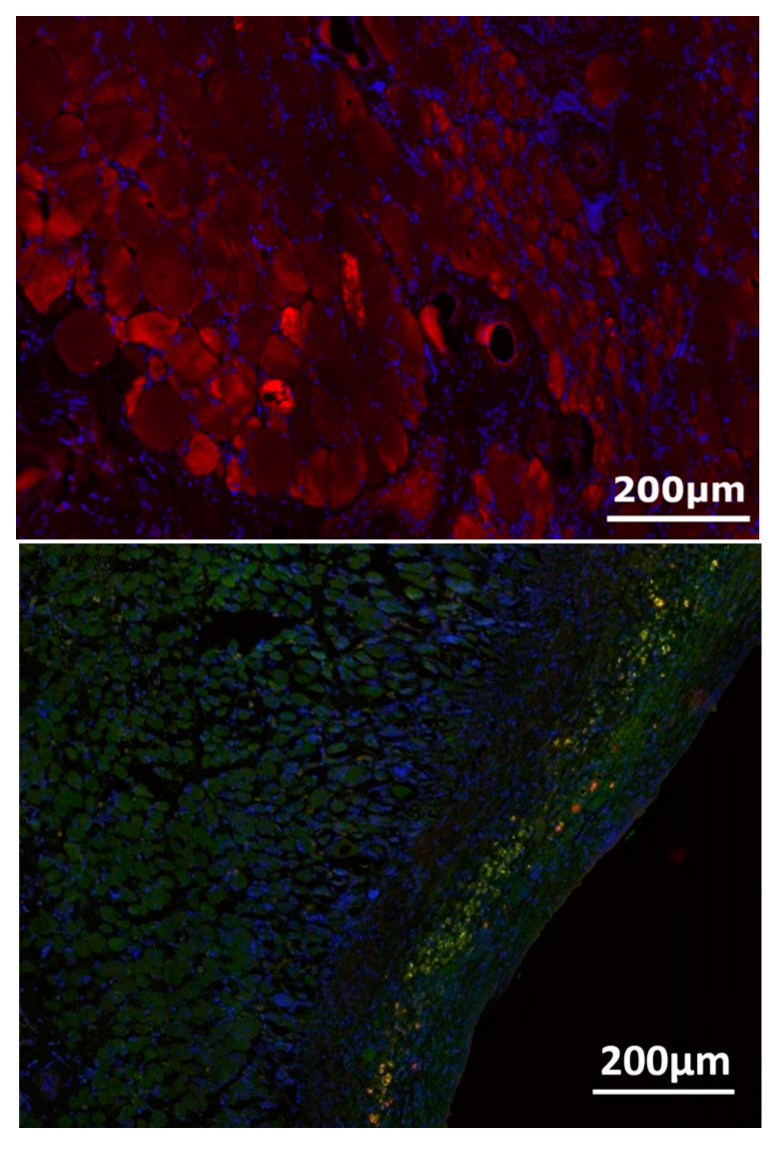
MHC staining (red) revealed irregularly arranged myofibrils in the transplanted muscle constructs (**top**). Double staining for synaptophysin (green) for nerve terminals and α-Bungarotoxin (red) for acetylcholine receptors showed neuromuscular junctions at the surface of the muscle near the epimysium after 2 weeks of transplantation and denervation (**bottom**).

MHC staining revealed irregularly arranged myofibrils in the transplanted muscle constructs (Figure 10). Double staining for synaptophysin for nerve terminals and α-Bungarotoxin for acetylcholine receptors showed some scattering of neuromuscular junctions at the surface of the muscle near the epimysium (Figure 10). Synaptophysin expression dominated clearly over acetylcholine receptors, which only appeared scarcely at the muscle surface.

### 3.5. Myofiber Morphology and Macrophage Invasion

**Figure 11:**
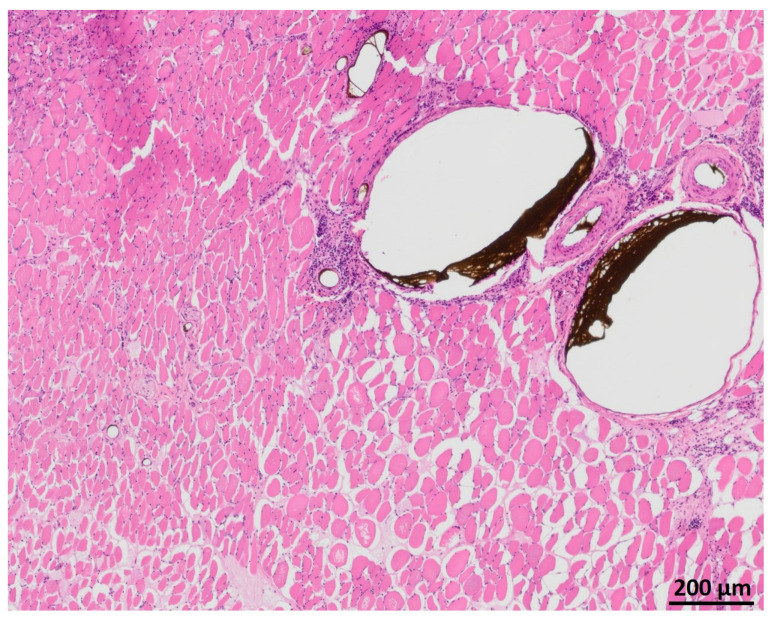
HE staining of the transplanted muscle showing partly well-preserved structures as well as partly inhomogenous and irregularly arrangement of the skeletal muscle.

**Figure 12:**
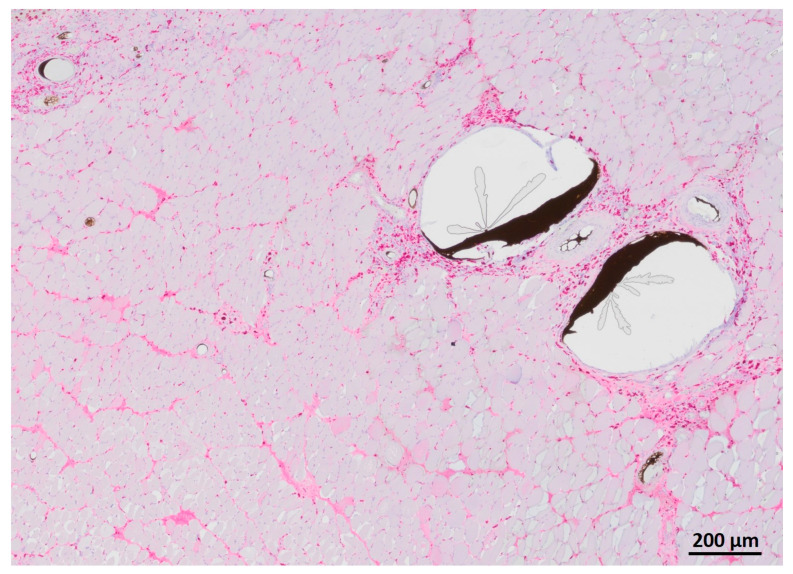
CD68 staining of transplanted muscle showing macrophages predominantly near the vessels filled with Microfil^®^.

HE staining showed partly well-preserved structures as well as partly inhomogenous and irregularly arrangement of the skeletal muscle similar to MHC staining (Figure 11). CD68 staining revealed invasion of macrophages, especially near the vessels (Figure 12). 

## 4. Discussion

In the present study, a novel rat model of heterotopic muscle transplantation in an isolation chamber was proposed. In situ muscle contraction properties before and after transplantation and denervation of the muscle were assessed. Furthermore, vessel distribution after 2 weeks of transplantation was characterized with both µCT and immunohistochemical analysis. 

Perfusion-decellularization of skeletal muscle has been successfully applied to the porcine rectus abdominis muscle [8]. Partial-thickness abdominal wall defects repaired with decellularized muscle scaffolds showed a higher amount of neo muscle tissue and neoangiogenesis compared to small intestinal submucosa, which showed the superiority of native tissue extracellular matrix for replacing muscle tissue [8]. Conconi et al. have shown the advantages of cell-seeded decellularized muscle matrices compared to matrices only, which were replaced by fibrous tissue shortly after implantation. However, after 60 days of implantation, even the cell–matrix constructs lost their full structural integrity, which could be explained by a missing axial vascularization [17]. The integration of a vascular axis results in prefabricated acellular matrices that can be transferred as flaps to repair defects [18]. Similarly, the goal of the present study was to set up a model which can be built on for whole muscle perfusion decellularization and subsequent recellularization and/or in vivo transplantation. For this purpose, a suitable muscle had to be chosen. There are various rodent models of muscle transplantation that have extensively described the anatomy and vascular supply of pectoralis major [19], gracilis [20], and quadriceps femoris muscles [21]. However, for the purposes of the present study, which is not solely a study of muscle transplantation, the medial head of the gastrocnemius muscle was chosen due to its single dominant vascular pedicle [22] and its spindle-like shape, which is beneficial for functional characterization and decellularization in a bioreactor as demonstrated recently [23]. In this study, gastrocnemius muscles were successfully perfusion-decellularized with their proximal tendinous parts being clamped in a bioreactor chamber, which enabled a continuous flow through the vascular system of the muscles while the muscles could be dynamically stretched [23]. As shown by others, repetitive stretching prevents denervation-induced muscle atrophy [24,25,26]. According to the aforementioned study, the mechanical stimulation in the reactor chamber preserved the effective decellularization [23]. Thus, this mechanism could be utilized for later recellularization of the constructs to preserve muscle functions. To set up baseline values for functionality and vascularization of such a de- and recellularized muscle construct, a native gastrocnemius muscle was transplanted within the present study. For minimizing external factors that could influence the transplanted muscle, e.g., extrinsic vascularization or fibrous tissue, an isolation chamber was designed that enclosed the muscle during the period of implantation. To enable tension-free closure of the wound, the size of the isolation chamber needed to be relatively small. This is also a reason for choosing the medial head of the gastrocnemius muscle of a smaller donor rat instead of a large muscle such as the quadriceps femoris muscle [21].

All force values decreased significantly after 2 weeks of transplantation. This finding is consistent with in vivo experiments that involved denervation of muscle, leading to muscle atrophy and a loss of isometric contraction force [27,28]. Microvascular flaps are denervated when transferred unless nerve coaptation is performed [29]. The effect of nerve coaptation (tibial nerve to a branch of the obturator nerve as described in the EPI-loop model [30]) would be the next step to analyze. Zhang et al. performed orthotopic transplantation of rat gracilis muscle with transection of the obturator nerve and subsequent neurorrhaphy of the nerve [31]. After 5–20 weeks after reinnervation, muscle function started to recover. Thus, it can be assumed that nerve coaptation would not have led to significant functional recovery in the present study where muscle transplantation was performed over a period of 2 weeks for establishing this novel model. Zhang et al. did not perform vessel anastomosis, which might have influenced the regeneration of nerve-muscle function. Cen et al. have shown that denervation impairs ischemic recovery of rat hindlimbs via impaired perfusion, lower capillary density, and narrower lumen of vessels, illustrating that angiogenesis depends on an intact peripheral nervous system [32]. In the present study, two different modalities were used to analyze vessel distribution. Both modalities showed that smaller vessels (0.1–20 µm in immunostains and 5–45 µm in µCT scans) were prevalent in the muscle constructs. While 2D immunosections only allowed for illustration of a few large vessels, 3D reconstruction of µCT scans enabled the quantification of cumulative lengths for different radius ranges. On the other hand, immunosections revealed smaller vessels more reliably since those vessels that were not filled with Microfil^®^ were also counted. The density of the capillary network and capillary distribution is crucial for muscle oxygenation and function [33]. The vast presence of capillaries as shown by immunosections and µCT implies that blood flow through the vascular pedicle is sufficient for supplying the muscles in the present study, at least at the current duration of denervation. Thus, a longer period of muscle implantation with or without nerve coaptation and its impact on muscle transplant function would be the logical next step of investigation. 

Unfortunately, muscle weight could not be determined after transplantation as explantation of the muscle took place after perfusion. This prohibited the normalization of peak force to muscle weight after transplantation. However, the significant decrease in absolute force values of more than 90% after transplantation shows that transplantation and denervation of the muscle lead to a loss of force, as would be expected. Fatigue index and passive stretch values did not change after transplantation which indicates that muscles remained vital after transplantation and did not develop a relevant amount of fibrosis after a period of 2 weeks. 

Synaptophysin, which is a glycoprotein in neurons and an essential component of synaptic vesicles, has shown stable expression even after different periods of denervation of gastrocnemius rat muscles [34] while postsynaptic acetylcholine receptors are known to decline with increased duration of denervation [35]. Furthermore, denervated muscles have shown disorganized and atrophic myofibers with inhomogenous HE staining [32]. Peripheral nerve injury, associated with Wallerian degeneration, is associated with inflammation and macrophage invasion [36]. This feature is in conjunction with the results of immunohistochemical staining of the transplanted muscles in the present study. One limitation of this study includes a missing reference muscle, which has not undergone transplantation, during immunohistochemical analysis. However, the study design did not enable us to collect additional native gastrocnemius muscles for formalin fixation since the muscles from the contralateral legs of the sacrificed rats were used for decellularization experiments [23]. Another limitation is the sole method of immunohistochemistry for analyzing myogenic markers and neuromuscular junctions. Evaluation of gene expression by PCR was not possible because whole muscle constructs were fixed in formalin for µCT analysis and paraffin embedding. The use of five animals in the present study is another limitation that sometimes resulted in high standard deviations. However, it was possible to show obvious differences in force values before and after muscle transplantation and to establish this model of whole muscle transplantation as a preliminary step towards transplantation of perfusion-decellularized muscle constructs as the purpose of the present study.

The previously mentioned shortcomings of this study, including missing nerve coaption, short follow-up period, and missing control group, will determine the goals for future studies, which will specifically evaluate the influence of motoric innervation and of an extended time period of 6–12 weeks on the functional outcome and the vascularization of the muscle constructs.

## 5. Conclusions

A novel model of successful whole muscle construct transplantation in an isolation chamber was established. Force values were characterized for native and transplanted muscles and vascularization was determined for transplanted muscles with both µCT and immunohistochemical methods. The findings of this study serve as a base for transplantation of perfusion-decellularized muscle constructs, but they can also serve as a base for other models, e.g., transplantation of functional muscle constructs for replacing denervated muscle tissue and analysis of their regeneration.

## Figures and Tables

**Table 1:** Animal data.

Rat	Donor Rat Weight (g)	Gastrocnemius Muscle Weight (g)	Recipient Rat Weight (g)	Ischemia Time (min)
1	285	1	410	120
2	240	0.87	440	100
3	260	0.94	400	85
4	290	1	370	90
5	320	1.03	410	90

**Table 2:** Force values before and after transplantation.

	Pre Transplantation	Post Transplantation
Peak twitch force (mN)	768.5 ± 266.6	34.7 ± 19.7 (**)
Time to peak twitch force (ms)	39.2 ± 6.0	28.1 ± 1.0 (*)
Time to 50% relaxation (twitch force) (ms)	22.5 ± 2.4	18.0 ± 3.7
Time to 100% relaxation (twitch force) (ms)	115.6 ± 8.3	124.5 ± 63.9
Maximum contraction rate (twitch) (N/s)	32.5 ± 8.6	1.9 ± 1.1 (**)
Maximum relaxation rate (twitch) (-N/s)	22.8 ± 7.5	1.4 ± 0.9 (**)
Maximum tetanic force (at 80 Hz) (mN)	2771 ± 772.7	28.6 ± 14.7 (**)
Maximum contraction rate (tetanus at 80 Hz) (N/s)	57.8 ± 14.7	1.0 ± 0.5 (**)
Time to peak force (tetanus at 80 Hz) (ms)	33.7 ± 7.1	19.6 ± 2.2 (*)
Maximum tetanic force (at 100 Hz) (mN)	2920 ± 981.9	32.1 ± 14.2 (**)
Maximum contraction rate (tetanus at 100 Hz) (N/s)	59.1 ± 10.5	1.1 ± 0.5 (***)
Time to peak force (tetanus at 100 Hz) (ms)	32.2 ± 4.6	19.7 ± 2.2 (**)
Maximum tetanic force (at 120 Hz) (mN)	2970 ± 1172	34.5 ± 13.4 (**)
Maximum contraction rate (tetanus at 120 Hz)	56.3 ± 19.7	1.2 ± 0.5 (**)
Time to peak force (tetanus at 120 Hz) (ms)	32.5 ± 2.8	19.6 ± 1.9 (***)

All values are mean ± SD; */**/*** indicate differences between muscles forces of gastrocnemius muscles pre and post transplantation (paired *t*-test); * *p* < 0.05; ** *p*< 0.01; *** *p* < 0.001.

**Table 3:** Vessel distribution depicted from µCT analysis.

Vessel Radius (µm)	Number of Vessels	Cumulative Length (mm)
5–45	4168 ± 1110	595.4 ± 112.1
55–95	3007 ± 1858	266.2 ± 122.9
105–145	1017 ± 794.1	155.7 ± 111.2
155–195	444.2 ± 372.2	96.3 ± 88.1
205–245	243.2 ± 188.4	61.5 ± 51.5
255–295	157 ± 90.7	45.6 ± 33.5
305–335	68.8 ± 42.9	24.9 ± 16.8

All values are the mean ± SD.

**Table 4:** Vessel distribution depicted from immunohistochemical analysis.

Vessel Radius (µm)	Number of Vessel Lumen
0.1–20	165.8 ± 109.5
20.1–40	17.4 ± 2.7
40.1–60	3.4 ± 1.5
60.1–80	1.8 ± 1.6
80.1–100	1.4 ± 0.9
100.1–120	0.4 ± 0.5
120.1–140	0.2 ± 0.4
140.1–160	0.2 ± 0.4
180.1–200	0.4 ± 0.5
220.1–240	0.4 ± 0.9
240.1–260	0.2 ± 0.4

All values are the mean ± SD.

## Data Availability

The data represented in this study are available on request from the corresponding author.
